# Supplementary material for: Potential Access to Emergency General Surgical Care in Ontario
Source: Int J Environ Res Public Health. 2022 Oct 22;19(21):13730. doi: 10.3390/ijerph192113730 (PMC9653868; doi:10.3390/ijerph192113730)
Supplement: Supplementary file 1 [file ijerph-19-13730-s001.zip › ijerph-1917662-supplementary.pdf]

Supplementary Material

Table S1. Population and associated land area of regions with potential access to any emergency general surgery care or a 24/7 emergency department.

		**Any Emergency General Surgery Care**					**24/7 Emergency Department**				
**Percentage with access within timeframe**		**15 min**	**30 min**	**45 min**	**60 min**	**90 min**	**15 min**	**30 min**	**45 min**	**60 min**	**90 min**
>80%	Population, n (%)*	11,064,852 (82)	12,820,808 (95)	13,100,546 (97)	13,178,284 (98)	13,247,785 (99)	10,218,664 (76)	12,678,983 (94)	13,088,815 (97)	13,158,067 (98)	13,234,250 (98)
	Land Area, km² (%)*	34,386 (3)	94,160 (10)	117,808 (12)	573,824 (58)	717,257 (73)	29,351 (3)	90,309 (9)	116,311 (12)	135,853 (14)	278,481 (28)
60.1-80	Population, n (%)	403,825 (3)	133,123 (1)	42,470 (<1)	14,272 (<1)	8,101 (<1)	411,956 (3)	153,399 (1)	35,252 (<1)	13,752 (<1)	15,835 (<1)
	Land Area, km² (%)	14,822 (2)	451,495 (46)	559,620 (57)	140,248 (14)	191,030 (19)	15,220 (2)	14,520 (1)	123,074 (12)	138,153 (14)	628,527 (64)
40.1-60	Population, n (%)	647,507 (5)	88,224 (1)	4,248 (<1)	12,728 (<1)	0	600,016 (4)	109,528 (1)	4,248 (<1)	12,728 (<1)	1,887 (<1)
	Land Area, km² (%)	457,953 (46)	133,316 (14)	16,470 (2)	45,268 (5)	N/A	20,386 (2)	119,471 (12)	16,470 (2)	45,268 (5)	758 (<1)
20.1-40	Population, n (%)	264,118 (2)	30,271 (<1)	10,247 (<1)	4,701 (<1)	4,078 (<1)	506,381 (4)	53,362 (<1)	17,751 (<1)	12,495 (<1)	4,078 (<1)
	Land Area, km² (%)	15,419 (2)	4,792 (<1)	41,835 (4)	2,758 (<1)	1,051 (<1)	15,773 (2)	457,618 (46)	478,571 (49)	440,830 (45)	1,051 (<1)
≤20%	Population, n (%)	1,056,329 (8)	364,205 (3)	279,120 (2)	226,646 (2)	176,667 (1)	1,699,614 (13)	441,359 (3)	290,565 (2)	239,589 (2)	180,581 (1)
	Land Area, km² (%)	464,143 (47)	302,961 (31)	250,991 (25)	224,625 (23)	77,385 (8)	905,994 (92)	304,806 (31)	252,297 (26)	226,619 (23)	77,906 (8)

*Percentage given in relation to total population and total land area where appropriate

Table S2. Population and associated land area of regions with potential access to a all emergency general surgery factors including a 24/7 operating room.

		24/7 Operating Room (All Factors)				
**Percentage with access within timeframe**		**15 min**	**30 min**	**45 min**	**60 min**	**90 min**
>80%	Population, n (%)*	9,313,610 (69)	12,050,424 (90)	13,036,672 (97)	13,133,554 (98)	13,209,148 (98)
	Land Area, km^2 (%)*	23,486 (2)	77,233 (8)	110,264 (11)	133,433 (14)	275,359 (28)
60.1-80	Population, n (%)	312,176 (2)	388,690 (3)	58,570 (<1)	17,539 (<1)	8,331 (<1)
	Land Area, km^2 (%)	9,025 (1)	13,805 (1)	126,163 (13)	139,617 (14)	191,790 (19)
40.1-60	Population, n (%)	540,703 (4)	278,392 (2)	9,275 (<1)	11,225 (<1)	9,632 (<1)
	Land Area, km^2 (%)	16,970 (2)	123,296 (12)	17,007 (2)	44,535 (5)	438,871 (44)
20.1-40	Population, n (%)	479,521 (4)	126,042 (1)	10,763 (<1)	6,204 (<1)	4,078 (<1)
	Land Area, km^2 (%)	14,806 (2)	23,737 (2)	41,984 (4)	3,491 (<1)	1,051 (<1)
≤20%	Population, n (%)	2,790,621 (21)	593,083 (4)	321,351 (2)	268,109 (2)	205,442 (2)
	Land Area, km^2 (%)	922,436 (93)	748,653 (76)	691,305 (70)	665,648 (67)	79,653 (8)

*Percentage given in relation to total population and total land area where appropriate
